# Glutamine mitigates murine burn sepsis by supporting macrophage M2 polarization through repressing the SIRT5-mediated desuccinylation of pyruvate dehydrogenase

**DOI:** 10.1093/burnst/tkac041

**Published:** 2022-12-30

**Authors:** Yuanfeng Zhu, Xiaoli Chen, Yongling Lu, Lin Xia, Shijun Fan, Qianying Huang, Xin Liu, Xi Peng

**Affiliations:** Clinical Medical Research Center, Southwest Hospital, Third Military Medical University (Army Medical University), Chongqing, 400038, China; Clinical Medical Research Center, Southwest Hospital, Third Military Medical University (Army Medical University), Chongqing, 400038, China; Clinical Medical Research Center, Southwest Hospital, Third Military Medical University (Army Medical University), Chongqing, 400038, China; Clinical Medical Research Center, Southwest Hospital, Third Military Medical University (Army Medical University), Chongqing, 400038, China; Clinical Medical Research Center, Southwest Hospital, Third Military Medical University (Army Medical University), Chongqing, 400038, China; Clinical Medical Research Center, Southwest Hospital, Third Military Medical University (Army Medical University), Chongqing, 400038, China; Clinical Medical Research Center, Southwest Hospital, Third Military Medical University (Army Medical University), Chongqing, 400038, China; Clinical Medical Research Center, Southwest Hospital, Third Military Medical University (Army Medical University), Chongqing, 400038, China; State Key Laboratory of Trauma, Burns and Combined Injury, Southwest Hospital, Third Military Medical University (Army Medical University), Chongqing, 400038, China

**Keywords:** Glutamine, SIRT5, Desuccinylation, PDH, Macrophages polarization, Burn, Sepsis

## Abstract

**Background:**

Alternative (M2)-activated macrophages drive the anti-inflammatory response against sepsis, a leading cause of death in patients suffering from burn injury. Macrophage M2 polarization is intrinsically linked with dominant oxidative phosphorylation (OXPHOS). Glutamine serves as a major anaplerotic source to fuel OXPHOS, but it remains unknown whether glutamine can modulate metabolic checkpoints in OXPHOS that favour M2 polarization. The study aims to explore whether glutamine essentially supports M2 polarization in IL-4-stimulated murine macrophages by sustaining the activity of PDH and whether glutamine augments macrophage M2 polarization and thus alleviates inflammation and organ injury in a murine burn sepsis model.

**Methods:**

To understand how glutamine promotes M2 activation in interleukin (IL-4)-treated murine macrophages, we detected glutamine-dependent M2 polarization and its relationship with the pyruvate dehydrogenase (PDH) complex by RT-PCR, flow cytometry and western blot. To explore how glutamine modulates PDH activity and thus supports M2 polarization, we compared the expression, phosphorylation and succinylation status of PDHA1 and then examined sirtuin SIRT5-dependent desuccinylation of PDHA1 and the effects of SIRT5 overexpression on M2 polarization by RT-PCR, flow cytometry and western blot. To determine whether glutamine or its metabolites affect M2 polarization, macrophages were cocultured with metabolic inhibitors, and then SIRT5 expression and M2 phenotype markers were examined by RT-PCR, flow cytometry and western blot. Finally, to confirm the in vivo effect of glutamine, we established a burn sepsis model by injecting Pseudomonas aeruginosa into burn wounds and observing whether glutamine alleviated proinflammatory injuries by RT-PCR, flow cytometry, western blot, immunofluorescent staining, hematoxylin-eosin staining and enzyme-linked immuno sorbent assay.

**Results:**

We showed that consumption of glutamine supported M2 activation in IL-4-treated murine macrophages by upregulating the activity of PDH. Mechanistically, glutamine did not affect the expression or alter the phosphorylation status of PDHA1 but instead downregulated the expression of SIRT5 and repressed SIRT5-dependent desuccinylation on PDHA1, which in turn recovered PDH activity and supported M2 polarization. This effect was implemented by its secondary metabolite α-ketoglutarate (αKG) rather than glutamine itself. Finally, we demonstrated that glutamine promoted macrophage M2 polarization in a murine burn sepsis model, thereby repressing excessive inflammation and alleviating organ injury in model mice.

**Conclusions:**

Glutamine mitigates murine burn sepsis by essentially supporting macrophage M2 polarization, with a mechanism involving the repression of the SIRT5-mediated desuccinylation of pyruvate dehydrogenase that replenishes OXPHOS and sustains M2 macrophages.

## Highlights

- Glutamine supports M2 polarization in IL-4 treated murine macrophages by sustaining PDH activity.
- Glutamine inhibits SIRT5-dependent desuccinylation on PDHA1 to maintain PDH activity.
- αKG, a glutamine metabolite, inhibits SIRT5 and supports M2 polarization.
- Glutamine alleviates inflammatory injury in murine burn sepsis by promoting macrophage M2 polarization.

## Background

Sepsis is a common complication post burn injury and the leading cause of mortality in burn patients [1,2]. Indeed, burn patients are more prone to developing sepsis than other traumatic diseases, which is ascribed to a severe loss of skin barrier causing uncontrolled burn wound infection and inducing a persistent hyperinflammatory status [3]. Macrophages are a ubiquitous group of innate immune cells in host tissues and compartments where they serve as gatekeepers to recognize and respond to infection and tissue injury [4]. Hyperactive macrophages are predominantly detected after burn injury, with increased production of tumour necrosis factor-α (TNF-α), interleukin 6 (IL-6) and IL-1. Many of these cytokines synergize with each other to induce a robust proinflammatory response, leading to the development of post-burn sepsis [1,5,6].

Macrophages exhibit a highly heterogeneous and plastic nature in response to environmental cues or in injured tissues [7]. Phenotypic polarization represents a typical form of macrophage plasticity, which is characterized by two extreme switches termed classically activated (M1) macrophages and alternatively activated (M2) macrophages [8]. M1 polarization is induced by microbial factors [e.g. lipopolysaccharide (LPS)] and proinflammatory mediators (e.g. interferon-γ) that confer host defence against infection or tumours. M2 polarization is activated by the increased production of anti-inflammatory cytokines (e.g. IL-4 and IL-13), favouring the transition into resolution of inflammation and tissue repair [9,10]. In parallel with external stimuli that govern phenotype skewing in macrophages, a fine-tuning intrinsic regulatory machinery may also critically orchestrate macrophage polarization and maintain the balance between immune response and tissue integrity [10–12]. Indeed, profound metabolic rewiring has emerged as both a distinguished signature and a critical inherent mechanism in macrophage polarization [13]. Typically, M1 macrophages rely on glycolysis for energy demand and produce metabolites such as citrate, succinate, lactate and nitric oxide to provoke inflammatory and antimicrobial reactions [13]. Conversely, intact oxidative phosphorylation (OXPHOS) is maintained in M2 macrophages, which not only meets the energy demand but also couples with other metabolic pathways (glutamine and polyamine metabolism, etc.) to promote the resolution of inflammation and tissue remodelling [12].

Glutamine is a major source of both carbon and nitrogen in metabolism and biosynthesis [14]. Typically, glutamine fuels the tricarboxylic acid (TCA) cycle by providing its main metabolite α-ketoglutarate (αKG) via a process termed glutaminolysis [14]. This metabolic feature makes glutamine a critical anaplerotic source that sustains an intact OXPHOS process for M2 polarization [12]. Indeed, recent studies have shown that glutamine accumulates in M2 macrophages due to enhanced uptake and synthesis [15,16]. Labelling studies have also confirmed the enrichment of ^13^C_5_-glutamine-labelled metabolites in the TCA cycle in IL-4-treated murine macrophages [17]. Furthermore, deprivation of extracellular glutamine may result in the reduction of M2 macrophages and the downregulation of M2 activation markers upon IL-4 stimulation [10,18]. These findings strongly imply an increasing demand for glutamine following IL-4 treatment, which supports the switch and sustainment of macrophage M2 polarization.

Although glutamine can directly provide intermetabolites to fuel the TCA cycle in M2 macrophages, glutamine or its metabolites may play additional roles in sustaining the TCA cycle and driving M2 polarization. For example, two recent studies demonstrated that glutamine activates αKG-dependent epigenetic regulation that upregulates M2 gene expression [10] or otherwise promotes uridine diphosphate *N*-acetylglucosamine synthesis to increase glycosylated modification of M2 marker proteins [18]. Strikingly, the latter study also reported that only one-third of all carbons in TCA cycle metabolites are derived directly from glutamine in M2 macrophages. Meanwhile, the gene set enrichment assay demonstrates a profound downregulation of the TCA cycle pathway in glutamine deprived M2 macrophages [18]. In this regard, a potential interplay may exist between glutamine and metabolic checkpoints in the TCA cycle to support M2 polarization, in addition to the already known effect of providing fuel for the process, or to upregulate M2 genes or proteins [18]. However, the linking mechanisms have not yet been elucidated.

The pyruvate dehydrogenase (PDH) complex occupies a central node in the TCA cycle by associating glycolysis with OXPHOS [19] through catalysing pyruvate oxidation [20]. Notably, the activity of PDH is impaired in M1 macrophages but maintained or even increased in M2 macrophages, suggesting that PDH may act as a metabolic checkpoint in macrophage M2 polarization [21]. Activation of PDH is modulated via transcriptional mechanisms or posttranslational modification, including phosphorylation, acetylation and succinylation [20,22]. Phosphorylation is a well-characterized mechanism of PDH inactivation that is catalysed by its upstream PDH kinases (PDKs). Indeed, PDK activation is widely demonstrated to repress M2 polarization and favour M1 activation by interrupting PDH activity and OXPHOS [21,23]. These findings further support PDH as a potential target for modulating macrophage M2 polarization in a metabolic manner, although it is not known whether it may become a target of glutamine. To this end, we explored in this study whether glutamine essentially supports M2 polarization in IL-4-stimulated murine macrophages by sustaining the activity of PDH. We also investigated how glutamine promotes PDH activation by examining both transcriptional and posttranslational mechanisms. Finally, we determined whether glutamine augments macrophage M2 polarization and thus alleviates inflammation and organ injury in a murine burn sepsis model.

## Methods

### Reagents

Dulbecco’s modified eagle’s medium (DMEM), glutamine-free DMEM, glutamine, IL-4 and LPS were purchased from Sigma Aldrich (St. Louis, MO, USA). Fetal bovine serum (FBS) was obtained from PAN Biotech (Bavaria, Germany). CPI-613, MC3482 and succinyl phosphonate (SP) were obtained from MedChemExpress (Monmouth Junction, NJ, USA). BLZ945 and L-α-aminoadipic acid (L-α-A) were purchased from APExBIO Technology LLC (Houston, TX, USA). CB-839 was obtained from Selleck Chemicals (Houston, TX). Co-immunoprecipitation (Co-IP) assay kit was purchased from Thermo Fisher Scientific (Rockford, IL, USA). Primary antibodies against arginase-1 (Arg1), CD206, PDH E1 component subunit alpha (PDHA1), phosphorylated PDHA1 (p-PDHA1), tubulin and IgG isotype control antibodies were purchased from Abcam (Cambridge, UK). Primary antibodies against pan-succinyllysine (Pan-succK) and pan-acetyllysine (Pan-acetK) were obtained from PTM BIO (Hangzhou, China). Primary antibodies against sirtuin SIRT5 and F4/80 and HRP-linked anti-mouse and anti-rabbit secondary antibodies were purchased from Cell Signaling Technology (Boston, MA, USA). Primary antibody against proliferating cell nuclear antigen (PCNA) was purchased from Proteintech Group (Rosemont, IL, USA). FITC and Cy3- labelled secondary antibodies were purchased from Beyotime Biotechnology (Shanghai, China).

### Animals

Wild-type BALB/c mice (male, 6–8 weeks) were purchased from SJA Laboratory Animal Co., Ltd (Hunan, China). All mice were housed under standard pathogen-free conditions with free access to water and food. Animal experiments were approved by the Institutional Animal Ethics Committee of the Third Military Medical University and performed in accordance with the national guidelines for animal welfare.

### Isolation of murine bone marrow derived macrophages

Murine bone marrow derived macrophages (BMDMs) were prepared as described [21]. Briefly, mice were sacrificed and the femur and tibia were dissected. After that, the bone marrow cavity was irrigated with 5 ml of normal saline (NS). The red blood cells were lysed, and the remaining bone marrow precursors were differentiated by culturing in DMEM containing 10% FBS and 50 ng/ml macrophage colony-stimulating factor (M-CSF) for 7 days. The purity of the BMDMs was determined by F4/80 staining via a flow cytometry assay. Cells with purity >95% qualified for subsequent experiments.

### Cell culture and treatment

Murine BMDMs and J774A.1 cells (ATCC, Manassas, VA, USA) were cultured in DMEM containing 10% FBS at 37°C in a humidified incubator supplemented with 5% CO_2_. In some experiments, BMDMs were cultured in glutamine-free DMEM medium for glutamine deprivation.

### Lentiviral overexpression of SIRT5

J774A.1 cells (2 × 10^5^ cells/ml) were seeded in 6-well plates and incubated for 2 h before transfection with a mouse SIRT5 overexpression lentivirus (Gene, Shanghai, China) (multiplicity of infection (MOI) = 20) for 24 h. Then cells were washed with fresh DMEM containing 10% FBS and further incubated for 48 h. Positive cells were screened with 5 μg/ml puromycin treatment for 24 h. The efficiency of SIRT5 overexpression was determined by real-time PCR (RT-PCR) and western blot.

### siRNA interference

J774A.1 cells (5 × 10^5^ cells/ml) were seeded in 6-well plates. Specific Small interfering RNA (siRNA) for PDHA1 and control siRNA (RiboBio Biotech, Guangzhou, China) were diluted with 30 μl of transfection buffer to a final concentration of 50 nM, mixed with 3 μl of transfection reagent (RiboBio Biotech) and added to the cultured cells for a 48 h incubation. The efficiency of siRNA interference against PDHA1 was determined by RT-PCR and western blot.

### Bacterial culture

*Pseudomonas aeruginosa* (*P. aeruginosa*) (ATCC27853) strain was inoculated in lysogeny broth medium and cultured at 37°C to the logarithmic phase (OD600 = 0.6–0.8) and then diluted to 7.5 × 10^4^ colony-forming unit (CFU)/ml before further use.

### Animal experiments

The mouse model of burn sepsis was established as previously described [24]. Briefly, mice were anaesthetized and the back skin was depilated. Then the mice were burned about 2 cm in diameter throughout the full thickness of skin (III°, about 15% total body surface area) using 90 °C water for 8 s. The scalded area was then immediately subcutaneously injected with *P. aeruginosa* (3.75 × 10^5^ CFU/kg body weight) to create a burn sepsis model and mice were resuscitated with NS (50 ml/kg) and kept warm in a 30°C incubator for 24 h. Subsequently, mice were intraperitoneally injected with 0.75 g/kg glutamine or an equal volume of NS every 24 h. Mouse back skin was immersed in a 37°C water bath for 8 s as a sham scald. In some experiments, mice received BLZ945 (200 mg/kg body weight) or vehicle (20% Captisol) by oral gavage once daily for 7 days prior to burn sepsis modelling. For sample analysis, the mice were sacrificed 24 and 72 h after glutamine injection. Peritoneal samples were collected by peritoneal lavage with 5 ml of NS. Peritoneal lavage fluid (PLF) and peritoneal macrophages were then isolated by centrifuge. Blood samples were collected by cardiac puncture. The whole right lobe of liver was eviscerated and sampled for histological, enzyme-linked immunosorbent assay (ELISA) and immunofluorescence examination. The serum samples were then used for detection of cytokines, alanine transaminase (ALT) and aspartate aminotransferase (AST). PLF samples were used for flow cytometry. The 7-day survival of burn sepsis mice was observed.

### Flow cytometry

Single-cell suspensions of the treated BMDMs and J774A.1 cells (2 × 10^5^/ml) or freshly isolated murine peritoneal macrophages were fixed in 10% paraformaldehyde and stained with PE-anti-CD11b, FITC-anti-F4/80, APC-anti-CD206 and APC-anti-CD80 (biolegend, San Diego, CA) at 4°C for 30 min. Fluorescence staining was detected on an ACEA NovoCyte flow cytometer (San Diego, CA, USA). The percentages of M2 macrophage (F4/80^+^CD11b^+^CD206^+^) or M1 macrophage (F4/80^+^CD11b^+^CD80^+^) populations were detected on an ACEA NovoCyte flow cytometer (San Diego, CA) and calculated using the Novo express software.

### PDH activity assay

The PDH activity in BMDMs and J774A.1 cells was detected using a PDH activity assay kit (Sigma-Aldrich, St. Louis, MO, USA). The absorbance was measured by a Varioskan flash multimode reader (Thermo Fisher Scientific).

### Intracellular glutamine measurement

Intracellular glutamine in BMDMs and J774A.1 cells was detected using a glutamine assay kit (Abcam, Cambridge, UK). The absorbance was measured by a Varioskan flash multimode reader.

### ALT and AST detection

Serum of burn sepsis mice was collected at the indicated time. ALT and AST concentrations were measured with assay kits (Jiancheng, Nanjing, China) according to the manufacturer’s instructions. The concentration of ALT and AST is indicated in Karman’s units (KU/ml).

### Real-time PCR assay

Total RNA was extracted from cell lysates using an RNasyMini Kit (Qiagen, Hilden, Germany) and reverse transcribed into cDNA with a ReverTra Ace qPCR RT Master Mix (TOYOBO, Osaka, Japan) following the operational instructions. The cDNA templates were mixed with iTaq Universal SYBR Green Supermix (Bio-rad, Hercules, CA, USA) and target genes primers (sequences listed in Table S1, see online supplementary material). Real-time PCR was performed using a Bio-Rad CFX96 Real-Time System.

### Co-IP assay

Dynabeads protein A/G (5 μg; Thermo Fisher Scientific) were mixed with 10 μg of anti-SIRT5 or anti-PDHA1 antibody (Santa Cruz Biotechnology, Heidelberg, Germany) and incubated at room temperature with rotation for 30 min. Then 250 μg of cytoplasmic proteins were added and incubated overnight at 4°C. The dynabeads were washed three times with washing buffer. The proteins bound to the dynabeads were eluted with acid elution buffer and then boiled at 100°C for 5 min in loading buffer and separated by SDS-PAGE. The proteins were transferred to a PVDF membrane and blocked with 5% bovine serum albumin for 1 h. The membrane was incubated with anti-PDHA1, anti-SIRT5 and anti-pan-succinyllysine primary antibodies (1:1000 dilutions) at 4°C overnight. The membrane was washed with TBST and incubated with an HRP-linked secondary antibody (1:1000 dilutions) for 1 h. After washing with TBST, the bands were visualized with a Super ECL plus western blotting Substrate kit (Bioground, Chongqing, China) and detected using a Vilber Fusion FX-EDGE imaging system (Paris, France).

### ELISA

Supernatants from cultured murine macrophages, serum and liver tissue homogenate of burn sepsis mice were collected. TNF-α, IL-6 and IL-10 concentrations were detected with ELISA kits (Thermo Fisher Scientific, Rockford, IL, USA) according to the manufacturer’s instructions.

### Western blotting

Cells were lysed with T-PER protein extraction reagent (Thermo Fisher Scientific) containing a protease and phosphatase inhibitors cocktail. Proteins were separated by SDS-PAGE and transfered to PVDF membranes. After blocking with 5% bovine serum albumin for 1 h, the membranes were incubated with primary antibodies (1:1000 dilutions) for Arg1, CD206, SIRT5, PDHA1, Pan-succK, Pan-acetK and tubulin at 4°C overnight, followed by incubation with HRP-linked secondary antibodies (1:2000 dilutions) at room temperature for 1 h. Chemiluminescence images were visualized using a Super ECL plus western blotting Substrate kit (Bioground biotechnology, Chongqing, China) and detected using a Vilber Fusion FX-EDGE imaging system (Grand Paris, France).

### Hematoxylin–eosin staining

Liver tissues extracted from normal and burn sepsis mice were fixed in 4% paraformaldehyde (Boster, Wuhan, China). Subsequently, the paraffin-embedded tissue sections were stained with hematoxylin and eosin and analyzed by a Zeiss light microscopy (Jena, Germany).

### Bimolecular fluorescence complementation detection

Plasmid expressing PDHA1 (0.5 μg) (pBIFC-VN173-PDHA1, where BIFC is bimolecular fluorescence complementation detection) and SIRT5 (pBIFC-VC155-SIRT5) (Miaoling plasmid, Hubei, China) were diluted with 25 μl of opti-MEM, and 0.75 μl of Lipofectamine 3000 was diluted with 25 μl of opti-MEM. The diluted plasmids were mixed with transfection reagents and incubated at room temperature for 20 min. After that, PDHA1 and SIRT5 plasmids-transfection reagent mixtures were added to 293 T cells (1 × 10^5^ cells/ml) alone or simultaneously and incubated in glass bottom dishes for 48 h. Fluorescence images were detected by LSM880 laser scanning confocal microscope (Zeiss, Oberkochen, Germany).

### Immunofluorescent assay

The liver tissues of mice were frozen and sectioned by a leica CM1950 frozen slicer (Weztlar, Germany). Then the samples were fixed by acetone and incubated with primary antibodies against F4/80 and CD206 (CST, MA, USA) or PCNA at 4°C overnight, followed by staining with FITC or Cy3-labelled secondary antibodies (Beyotime, China) for 1 h and counterstaining with DAPI (Beyotime) for 5 min. Fluorescent images were acquired by LSM880 laser scanning confocal microscope.

### Statistical analysis

The quantitative data are expressed as mean plus standard deviation. Shapiro–Wilk Normality test was performed to determine data distribution. Student’s t-test was used to compare the data of two groups. Comparisons for multiple groups were performed using one-way ANOVA with *post hoc* Bonferroni corrections. *P*-value < 0.05 was considered statistically significant.

**Figure 1:**
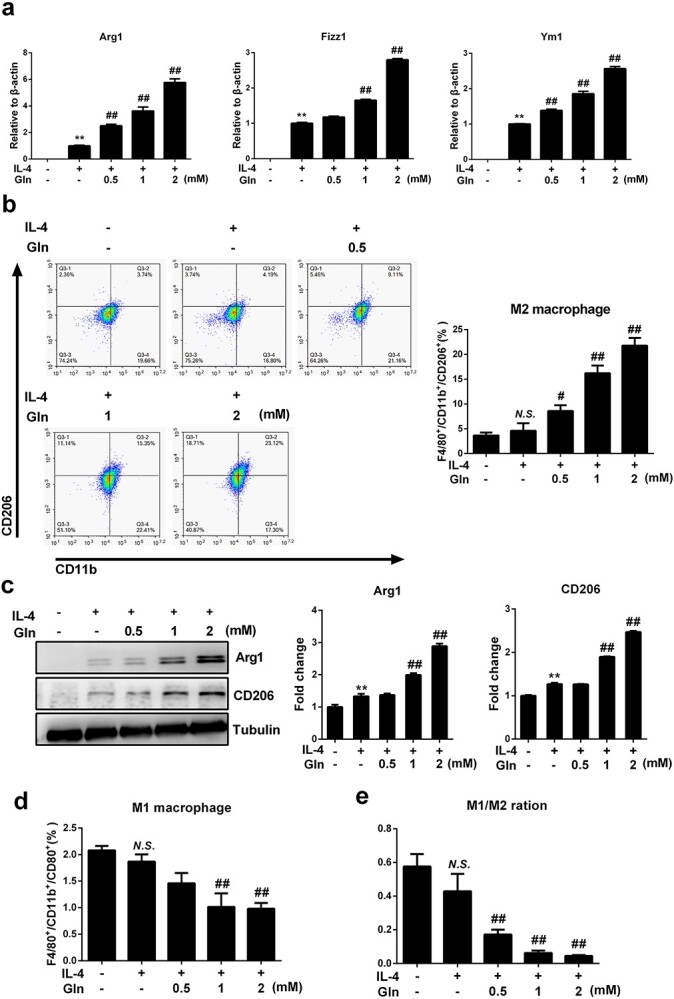
Glutamine (Gln) supplement augments macrophage M2 polarization in IL-4 treated BMDMs. (**a**) BMDMs were treated with 50 ng/ml IL-4 alone or together with gradient concentrations of glutamine (Gln, 0.5–2 mM) for 24 h. mRNA expression of Arg1, Fizz1 and Ym1 was detected by RT-PCR (**b**). F4/80^+^/CD11b^+^/CD206^+^ cells were detected by flow cytometry (**c**). Protein expression of Arg1 and CD206 was detected by western blot. F4/80^+^/CD11b^+^/CD80^+^ cells were detected by flow cytometry (**d**). The ratio of M1 and M2 macrophages was detected by flow cytometry (**e**). ^*^^*^p < 0.01, *N.S*. no significance *vs* treatment without glutamine and IL-4. #*p* < 0.05, ##*p* < 0.01 *vs* IL-4 treatment alone. *n* = 3. *BMDMs* bone marrow derived macrophages, *IL* interleukin

## Results

### Glutamine supports macrophage M2 polarization in IL-4-treated BMDMs

**Figure 2:**
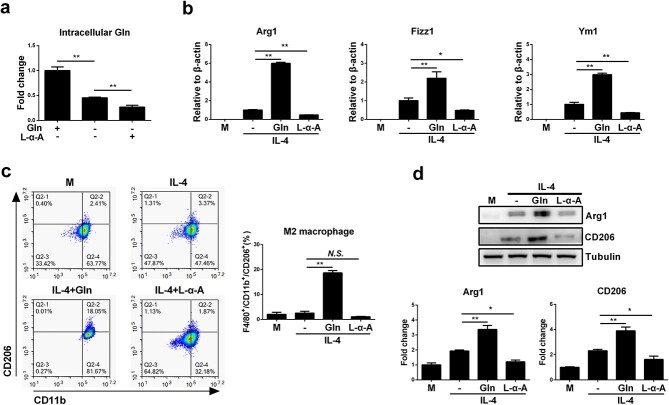
Intracellular deprivation of glutamine (Gln) inhibits M2 polarization of macrophages. (**a**) BMDMs were supplemented with Gln (2 mM) or starved of Gln or further treated with L-α-Aminoadipic acid (L-α-A, 200 μM) for 24 h. Intracellular Gln was detected. (**b**) BMDMs were treated with IL-4 (50 ng/ml) alone or together with Gln (2 mM) or further with L-α-A (200 μM) for 24 h. mRNA expression of Arg1, Fizz1 and Ym1 was detected by RT-PCR. (**c**) F4/80^+^/CD11b^+^/CD206^+^ cells were detected by flow cytometry. (**d**) Protein expression of Arg1 and CD206 was detected by western blot. ^*^p < 0.05, ^*^^*^p < 0.01. *N.S*. no significance. *n* = 3. *BMDMs* bone marrow derived macrophages, *IL* interleukin

**Figure 3:**
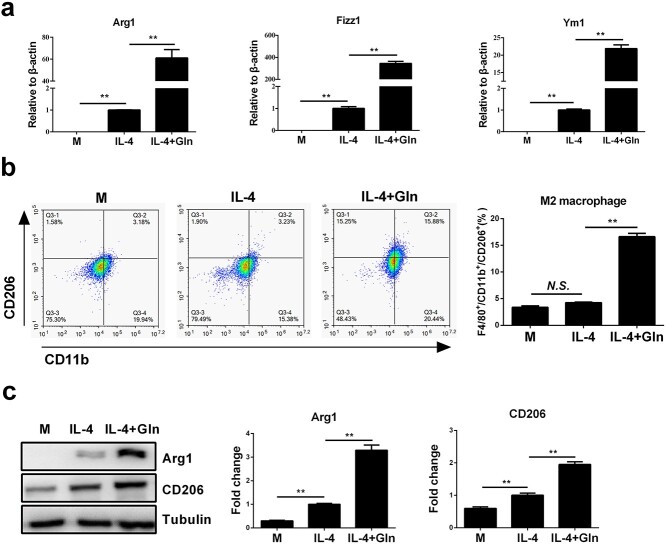
Glutamine (Gln) supplementation augments macrophage M2 polarization in IL-4-treated J774A.1 cells. (**a**) J774A.1 cells were treated with 50 ng/ml IL-4 alone or together with 2 mM Gln for 24 h. mRNA expression of Arg1, Fizz1 and Ym1 was detected by RT-PCR. (**b**) F4/80^+^/CD11b^+^/CD206^+^ cells were detected by flow cytometry. (**c**) Protein expression of Arg1 and CD206 was detected by western blot. ^*^^*^p < 0.01. *N.S*. no significance. *n* = 3. *IL* interleukin

To investigate whether extracellular glutamine supplementation is required for macrophage M2 polarization, murine BMDMs were treated with glutamine alone or together with IL-4, a classical cytokine that skews macrophages towards an M2 phenotype. Although glutamine alone did not promote M2 polarization in BMDMs (Figure S1, see online supplementary material), glutamine induced pronounced upregulation of M2-specific marker genes, including Arg1, found in inflammatory zone 1 (Fizz1) and Ym1, in IL-4-treated BMDMs in a dose-dependent manner (Figure 1a). Consistently, glutamine significantly increased the ratio of the F4/80^+^CD11b^+^CD206^+^ population of BMDMs and upregulated the plasma levels of Arg1 and CD206, both of which are protein markers in M2-polarized macrophages (Figure 1b, c). Further experiments showed that IL-4 had no ability to promote M1 polarization of macrophages while further glutamine treatment did not alter macrophage M1 polarization either (Figure 1d). Furthermore, the M1/M2 ratio in response to IL-4 was gradually reduced with elevated glutamine concentration, which is probably attributable to the increase in M2 macrophages (Figure 1e). Extracellular glutamine deprivation significantly decreased the intracellular levels of glutamine, and the use of L-α-A, an intrinsic glutamine synthase inhibitor, further reduced the intracellular glutamine concentration (Figure 2a). As expected, the use of L-α-A further inhibited the M2 polarization of macrophages induced by IL-4 (Figure 2b–d). Glutamine was also confirmed to augment macrophage M2 activation in IL-4-treated J774A.1 macrophages (Figure 3a–c). Together, these results indicate that glutamine may play an important role in supporting M2 polarization in macrophages.

### Glutamine enhances macrophage M2 polarization by increasing the activity of PDH

**Figure 4:**
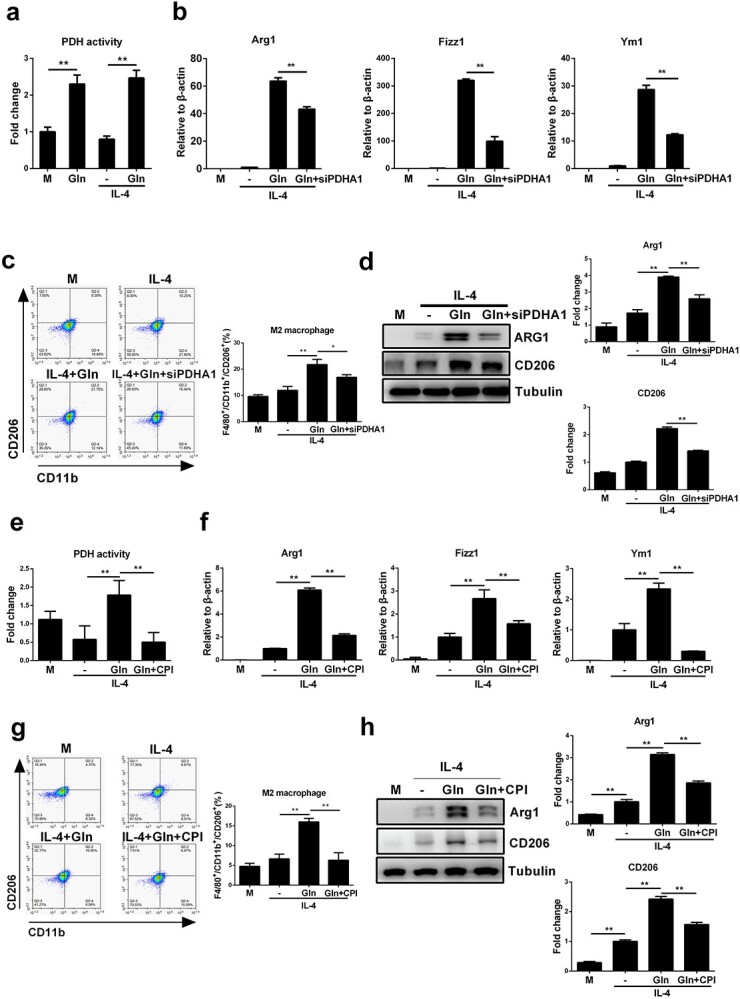
Glutamine (Gln) enhances macrophage M2 polarization by increasing the activity of PDH. (**a**) BMDMs were treated with 2mM Gln alone, 50ng/ml IL-4 alone or IL-4 together with Gln for 24 h. PDH activity was detected by absorbance detection. (**b**) J774A.1 cells were transfected with PDHA1 siRNA (siPDHA1) for 48 h and then treated with 50 ng/ml IL-4 alone or further with 2 mM Gln for 24 h. mRNA expression of Arg1, Fizz1 and Ym1 was detected by RT-PCR. (**c**) F4/80+/CD11b+/CD206+ cells were detected by flow cytometry. (**d**) Protein expression of Arg1 and CD206 was detected by western blot. (**e**–**h**) BMDMs were treated with 50 ng/ml IL-4 alone or together with 2 mM Gln or further treated with 10 μM CPI-613 (CPI) for 24 h. PDH activity was detected by absorbance detection (e). mRNA expression of Arg1, Fizz1 and Ym1 was detected by RT-PCR (f). F4/80^+^/CD11b^+^/CD206^+^ cells were detected by flow cytometry (g). Protein expression of Arg1 and CD206 was detected by western blot (h). ^*^p < 0.05, ^*^^*^p < 0.01. *N.S*., no significance. *n* = 3. *BMDMs* bone marrow derived macrophages, *IL* interleukin

We next addressed whether glutamine synergizes with IL-4 to regulate PDH, a metabolic checkpoint in the TCA cycle that sustains the activity of OXPHOS for M2 macrophages [20]. Glutamine enhanced the activity of PDH no matter whether IL-4 was given or not. In contrast, whereas IL-4 alone did not alter the enzymatic activity of PDH, concurrent treatment with IL-4 and glutamine significantly upregulated PDH activation (Figure 4a). To verify that glutamine boosts M2 macrophages by targeting PDH, we downregulated the expression of PDHA1, a key subunit of the PDH complex, by siRNA interference performed in J774A.1 macrophages (Figure S2, see online supplementary material). Knockdown of PDHA1 significantly inhibited macrophage M2 polarization despite glutamine and IL-4 co-treatment, as demonstrated by reduced mRNA expression of Arg1, Fizz1 and Ym1 (Figure 4b), a decreased percentage of M2 macrophages (Figure 4c) and downregulated protein levels of Arg1 and CD206 (Figure 4d). To confirm the above findings, we further treated BMDMs with CPI-613, a chemical inhibitor of PDH. Similar to the results obtained using siRNA against PDHA1, CPI-613 treatment abolished PDH activation (Figure 4e) and disrupted M2 polarization in BMDMs (Figure 4f–h). Collectively, these results indicate that PDH may function as a metabolic checkpoint by which glutamine facilitates M2 polarization in IL-4-treated macrophages.

### Glutamine promotes PDH activity without upregulating its expression or affecting its protein phosphorylation

**Figure 5:**
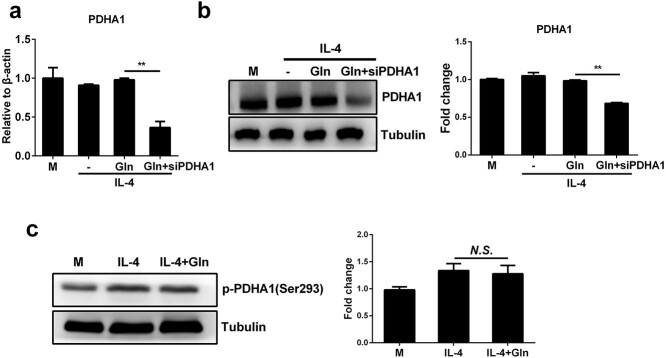
Glutamine (Gln) promotes PDH activity without upregulating its expression or affecting its protein phosphorylation. (**a**,**b**) J774A.1 cells were transfected with PDHA1 siRNA (siPDHA1) for 48 h. Then cells were treated with 50 ng/ml IL-4 alone or together with 2 mM Gln for 24 h. mRNA (a) and protein (b) expression of PDHA1 were detected by RT-PCR and western blot, respectively. (**c**) BMDMs were treated with 50 ng/ml IL-4 alone or together with 2 mM Gln for 24 h. The phosphorylation of PDHA1 (p-PDHA1) was detected by western blot. ^*^^*^p < 0.01. *N.S*. no significance. *n* = 3. *BMDMs* bone marrow derived macrophages, *IL* interleukin

To address how glutamine modulates the activity of PDH, we examined the expression of PDH in BMDMs when glutamine was supplemented. As shown by RT-PCR and western blot analysis, glutamine did not upregulate the mRNA or protein level of PDHA1 (Figure 5a, b). Therefore, glutamine may not promote PDH activation by increasing its expression but instead by affecting the posttranslational regulation of PDH. Previous studies have suggested that phosphorylation is a major posttranslational repressive mechanism that tightly regulates the activity of PDH [20]. However, we observed that glutamine had no effect on the phosphorylation status of PDHA1 on Ser293, thereby excluding the possibility that glutamine increases PDH by inhibiting its phosphorylation (Figure 5c). Together, these data demonstrate that glutamine may enhance PDH activation in IL-4-treated macrophages by mechanisms other than upregulating its expression or altering its phosphorylation status.

### Glutamine inhibits SIRT5 expression and interferes with SIRT5-dependent protein desuccinylation on PDH to promote macrophage M2 polarization

**Figure 6:**
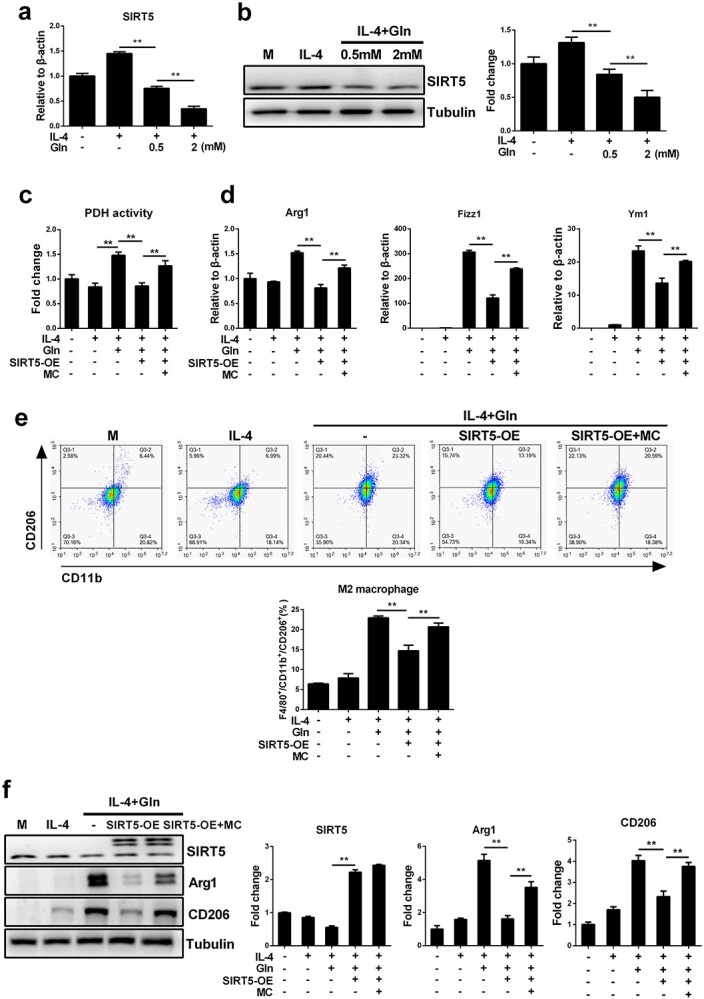
Glutamine (Gln) promotes PDH activity and enhances macrophages M2 polarization by inhibiting SIRT5. (**a**,**b**) Control J774A.1 cells and SIRT5-overexpressing (SIRT5-OE) J774A.1 cells were treated with 50 ng/ml IL-4 alone or together with Gln (0.5 and 2 mM) for 24 h. mRNA (a) and protein (b) levels of SIRT5 were detected by RT-PCR and western blot, respectively. (**c**) Control J774A.1 cells and SIRT5-OE J774A.1 cells were treated with 50 ng/ml IL-4 alone or further with 2 mM Gln or 50 μM MC3482 (MC) for 24 h. PDH activity was detected by absorbance detection. (**d**–**f**) Control J774A.1 cells and SIRT5-OE J774A.1 cells were treated with 50 ng/ml IL-4 alone or together with 2 mM Gln or further treated with 50 μM MC3482 (MC) for 24 h. mRNA expression of Arg1, Fizz1 and Ym1 was detected by RT-PCR (d). F4/80^+^/CD11b^+^/CD206^+^ cells were detected by flow cytometry (e). Protein expression of SIRT5, Arg1 and CD206 was detected by western blot (f). ^*^^*^p < 0.01. *n* = 3. *IL* interleukin

The activity of PDH could be negatively regulated by its desuccinylation, which is primarily mediated by mitochondrial SIRT5 [22]. Therefore, we next sought to determine whether glutamine sustains the activity of PDH by repressing SIRT5-dependent desuccinylation. As expected, glutamine supplementation significantly inhibited the upregulation of SIRT5 at both the mRNA and protein levels in IL-4-treated BMDMs (Figure 6a, b). Then, SIRT5 overexpression was induced in J774A.1 macrophages via lentiviral transfection (Figure S3a–c, see online supplementary material). SIRT5-overexpressing J774A.1 macrophages were also treated with MC3482, a SIRT5 desuccinylase inhibitor, to interfere with protein desuccinylation. SIRT5 overexpression abolished the enhanced PDH activation induced by glutamine, whereas additional MC3482 treatment restored its activity (Figure 6c). In addition, SIRT5-overexpressing macrophages demonstrated a pronounced disruption of M2 polarization upon glutamine and IL-4 co-treatment, which was also recovered by further MC3482 treatment (Figure 6d–f).

**Figure 7:**
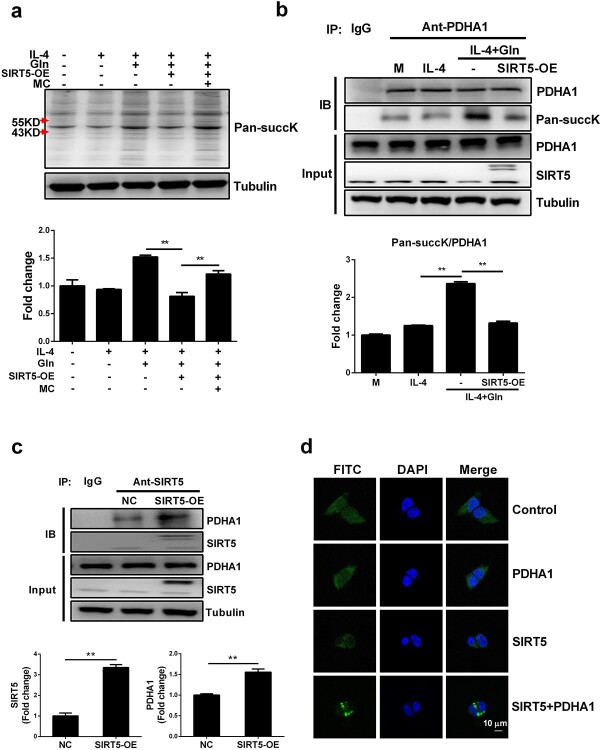
Glutamine (Gln) regulated PDHA1 succinylation modification by inhibiting the desuccinylation modification activity of SIRT5. (**a**) Control J774A.1 cells and SIRT5-OE J774A.1 cells were treated with 50 ng/ml IL-4 or IL-4 with 2 mM Gln or further treated with 50 μM MC3482 (MC) for 24 h. Pan-succinyllysine (Pan-succK) was detected by western blot. (**b**) Control J774A.1 cells and SIRT5-OE J774A.1 cells were treated with 50 ng/ml IL-4 or IL-4 with 2 mM Gln. PDHA1 protein was precipitated by immunoprecipitation using PDHA1 antibody and the succinylation modification of PDHA1 was detected by western blot using a Pan-succK antibody. (**c**) The amount of PDHA1 conjugated with SIRT5 was detected by co-immunoprecipitation assay. (**d**) pBIFC-VN173-PDHA1 (PDHA1) and pBIFC-VC155-SIRT5 (SIRT5) plasmids were transfected separately or together into 293 T cells for 48 h. Fluorescence images were detected by Ziss LSM880 laser scanning confocal microscope. Scale bar, 20 µm. ^*^^*^p < 0.01. *n* = 3. *IL* interleukin

We next examined how glutamine interrupts PDH desuccinylation driven by SIRT5. Pansuccinylation detection revealed that proteins in the molecular weight range 43–55 kDa, which includes PDHA1 (43 kDa), were increasingly succinylated by glutamine supplementation. Overall protein succinylation was suppressed by SIRT5-overexpression (SIRT5-OE) but reversely upregulated by MC3482, indicating that SIRT5 affects this process (Figure 7a). To clarify whether there was a direct succinylation modification of PDHA1 by SIRT5, immunoprecipitation was performed via an anti-PDHA1 antibody and a pansuccinylation antibody. As expected, glutamine significantly enhanced the succinylation modification of PDHA1, whereas SIRT5 overexpression disrupted this effect (Figure 7b). We next examined the direct interaction between SIRT5 and PDHA1 that may explain the regulation of succinylation. PDHA1 protein was shown to directly precipitate with SIRT5 and precipitated more in SIRT5-overexpressing cells (Figure 7c). The BIFC assay also showed that 293 T cells displayed an enhanced fluorescence signal only when co-transfected with pBIFC-VN173-PDHA1 and pBIFC-VC155-SIRT5, which further suggests a direct interaction between SIRT5 and PDHA1 (Figure 7d). Collectively, these results indicate that glutamine suppresses the expression of SIRT5, thereby inhibiting its ability to desuccinylate PDHA1, which in turn promotes PDH activity and enhances macrophage M2 polarization.

### Glutamine increases macrophage M2 polarization via its metabolite α-KG

**Figure 8:**
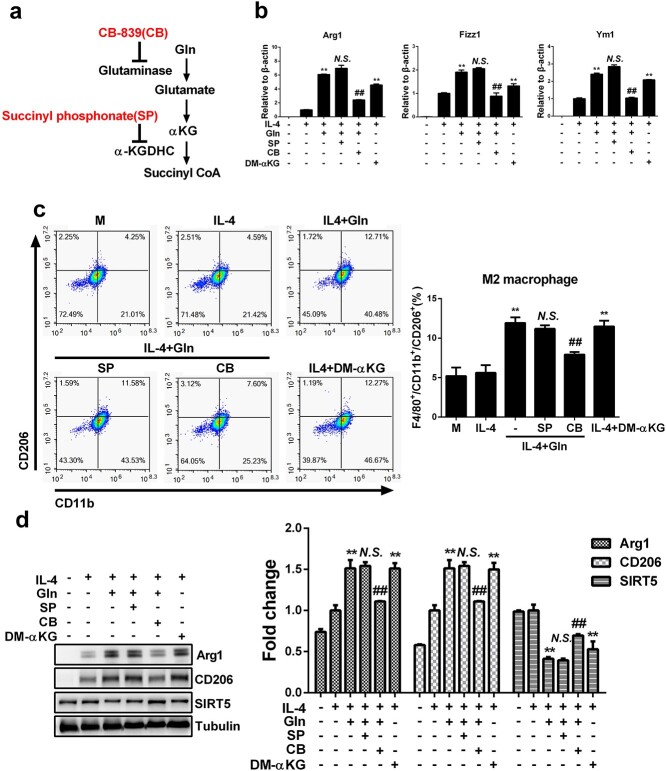
Glutamine (Gln) increases macrophage M2 polarization by its metabolite α-ketoglutarate (αKG). (**a**) A metabolic flowchart of Gln and the targets of inhibitors. (**b**–**d**) BMDMs were treated with 50 ng/ml IL-4, IL-4 with 2 mM dimethyl α-ketoglutarate (DM-αKG) or IL-4 with 2 mM Gln and further treated with 10 μM CB-839 (CB) or 50 μM succinyl phosphonate (SP) for 24 h. mRNA expression of Arg1, Fizz1 and Ym1 was detected by RT-PCR (b). F4/80^+^/CD11b^+^/CD206^+^ cells were detected by flow cytometry (c). Protein expression of Arg1, CD206 and SIRT5 was detected by western blot (d). ^*^^*^P < 0.01 *vs* IL-4, ##*p* < 0.01, *N.S*., no significance *vs* IL-4 + Gln. *n* = 3. *BMDMs* bone marrow derived macrophages, *IL* interleukin

Glutamine is catabolized into glutamate and then αKG and succinyl coenzyme A (succinyl CoA) to fuel the TCA cycle and modulate macrophage activation and the regulation of energy metabolism [10,25]. To investigate whether glutamine inhibits SIRT5 expression and promotes macrophage M2 polarization via its metabolites, CB-839 and SP were introduced to inhibit the production of αKG and succinyl CoA, while dimethyl α-ketoglutarate (DM-αKG), a cell-permeable analogue of αKG, was also used to directly examine the effect of αKG (Figure 8a). Whereas CB-839 markedly downregulated the expression of M2 marker genes (Figure 8b), decreased the ratios of M2 macrophages (Figure 8c) and inhibited the protein levels of M2 marker proteins (Figure 8d) in IL-4-treated macrophages with glutamine supplementation, SP had no effect on macrophage M2 polarization (Figure 8b–d). Furthermore, the decreased SIRT5 expression due to glutamine treatment was also inhibited by CB-839 but was not affected by SP (Figure 8d). Interestingly, DM-αKG could also induced pronounced macrophage M2 polarization and inhibited the expression of SIRT5 in IL-4-treated macrophages(Figure 8b–d). Therefore, the metabolite of αKG, instead of glutamine itself or the further metabolite succinyl CoA, may play an important role in mediating glutamine-dependent M2 polarization in macrophages.

### Glutamine alleviates murine burn sepsis injury by promoting macrophage M2 polarization

**Figure 9:**
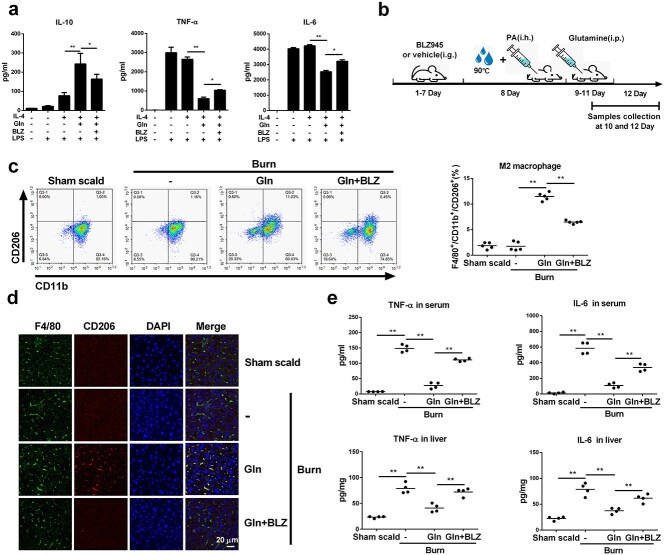
Glutamine (Gln) promoted M2 polarization of macrophages and reduced inflammation in burn sepsis mice. (**a**) Murine BMDMs were treated with 50 ng/ml IL-4, or IL-4 with 2 mM Gln or further treated with 10 μM BLZ-945 (BLZ) for 24 h. Then cells were stimulated with 20 ng/ml LPS for 24 h. Supernatant IL-10, TNF-α and IL-6 were detected by ELISA (*n* = 3). (**b**–**e**) Wild-type BALB/c mice were pretreated with BLZ945 (BLZ) for 7 days, then intraperitoneally injected with normal saline (Burn) or 0.75 g/kg Gln every 24 h after the burn sepsis model was created. Serum, peritoneal fluid and liver tissue were collected 1 day after Gln injection (b). F4/80^+^/CD11b^+^/CD206^+^ cells in peritoneal fluid were detected by flow cytometry (c) (*n* = 5). Immunofluorescence staining of liver tissue for F4/80 and CD206 was detected by ZISS LSM880 laser scanning confocal microscope (d). TNF-α and IL-6 in serum and liver tissue homogenate were detected by ELISA assay (e) (*n* = 4). ^*^^*^p < 0.01. Scale bar, 20 μm. *i.g*. intragastric administration, *i.h*. hypodermic injection, *i.p*. intraperitoneal injection. *BMDMs* bone marrow derived macrophages, *IL* interleukin, *TNF-α* tumor necrosis factor-α, *LPS* lipopolysaccharide

**Figure 10:**
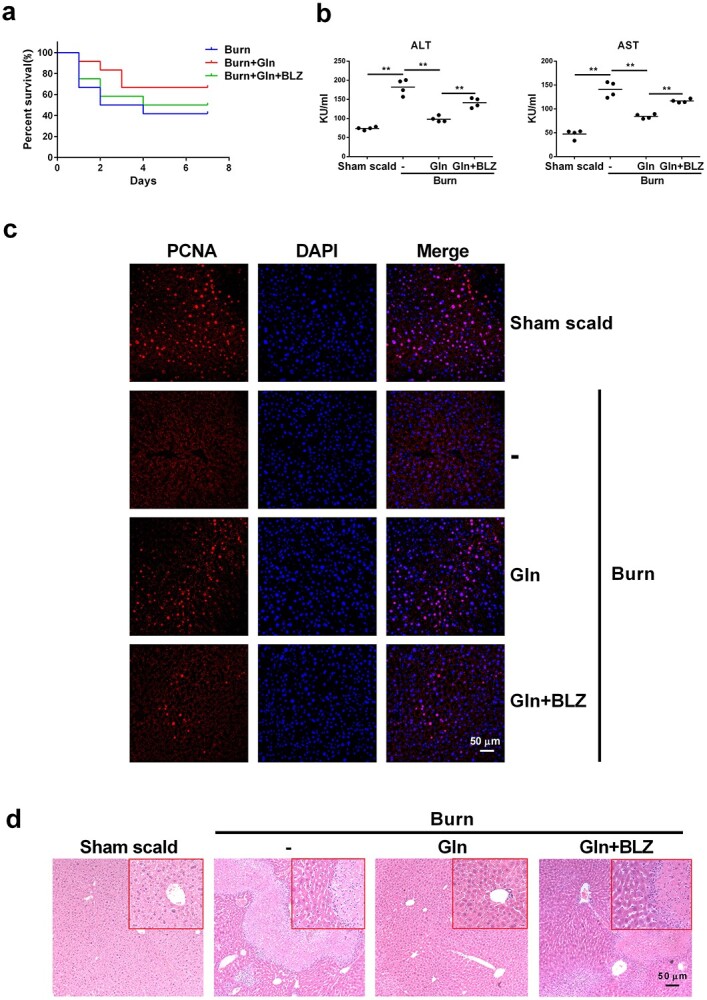
Glutamine (Gln) alleviates burn sepsis and organ damage. (**a**–**d**) Wild-type BALB/c mice were pretreated consecutively with BLZ945 (BLZ) for 7 days, then intraperitoneally injected with normal saline (Burn) or 0.75 g/kg Gln 24 h after the burn sepsis model was created. The survival of the mice was observed for 7 days (**a**) (*n* = 10). Serum was collected 1 day after Gln injection and the levels of ALT and AST were detected (**b**) (*n* = 4). Liver tissues were collected 3 days after Gln injection. The immunofluorescence of proliferating cell nuclear antigen (PCNA) in liver tissue was detected (**c**). Histopathological examination of liver was performed by hematoxylin and oesin (HE) staining (**d**). ^*^^*^p < 0.01. *N.S*. no significance. Scale bar, 50 μm. *ALT* alanine transaminase, *AST* aspartate aminotransferase

M2 macrophages play an important role in controlling excessive inflammation in sepsis [11,26]. To verify whether glutamine attenuates the proinflammatory reactions in burn sepsis, BMDMs were treated with IL-4 with or without glutamine and then stimulated with LPS. The presence of glutamine significantly augmented the ability of IL-4 to increase the production of the anti-inflammatory cytokine IL-10 while at the same time inhibiting proinflammatory TNF-α and IL-6 secretion (Figure 9a). BLZ945, a CSF-1R inhibitor that effectively inhibits M2 polarization of macrophages [27], disrupted the effect of glutamine to upregulate IL-10 and downregulate TNF-α and IL-6 production (Figure 9a). We next created a burn sepsis model in mice and examined the efficacy of glutamine administration with or without BLZ945 pretreatment (Figure 9b). A significant increase in M2 macrophages was detected in PLF or liver tissue upon glutamine injection, which was partly abolished when BLZ945 was preinjected to dampen macrophage M2 polarization (Figure 9c, d). Accordingly, the elevation of TNF-α and IL-6 in serum and liver homogenates of burn sepsis mice was markedly prohibited by glutamine treatment, which was also reversed by BLZ945 (Figure 9e). In the survival analysis, glutamine treatment had a tendency to increase the survival rates (from 41 to 66%) and delayed the median death time (from 3 days to >7 days) of the burn sepsis mice. This effect was abolished by further use of BLZ945 that inhibited M2 polarization of macrophages (Figure 10a). Liver function evaluation indicated that burn sepsis induced significantly increased levels of ALT and AST in serum (Figure 10b), decreased the expression of PCNA in hepatic cells (Figure 10c), and revealed the typical histological appearance of liver injuries in mice, as shown by hepatocyte swelling and remarkable tissue necrosis (Figure 10d). Glutamine significantly reduced serum ALT and AST while enhancing the expression of PCNA in hepatic cells and markedly reducing these pathological changes. In addition, further treatment with BLZ945 worsened liver injury in model mice (Figure 10b–d). Collectively, these results indicate that glutamine suppresses inflammation and reduces organ injury in murine burn sepsis by promoting M2 polarization of macrophages in mice.

## Discussion

**Figure 11:**
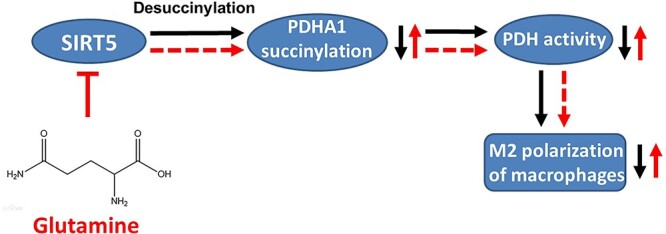
Schematic model depicting a hypothetical mechanism of glutamine promoting M2 polarization in macrophages

Severe burns are inevitably accompanied by serious complications, such as wound infection and sepsis [1,5,6]. Metabolic alteration is fundamental to macrophage polarization that orchestrates inflammation control in burn sepsis [25,28]. Glutamine controls metabolic remodelling and switches macrophage polarization by replenishing essential intermetabolites in OXPHOS [25]. However, whether glutamine can directly modulate key checkpoints in the TCA cycle that link metabolism with macrophage phenotyping is not clearly understood. Here, we reveal that glutamine supports M2 polarization in IL-4-treated murine macrophages by sustaining the activity of PDH, a checkpoint enzyme in the TCA cycle. This effect is mediated by its metabolite αKG, which inhibits SIRT5 expression and SIRT5-dependent desuccinylation on PDHA1, thereby maintaining PDH activity and allowing an intact OXPHOS process that supports M2 polarization. We also demonstrate that glutamine prevents excessive inflammation and reduces organ damage by promoting macrophage M2 polarization in a murine burn sepsis model, suggesting its therapeutic competence in postburn syndromes such as infection and sepsis. A schematic diagram is presented in Figure 11.

Glutamine is abundantly distributed in the body and serves as a primary carbon and nitrogen source [29]. Given this metabolic feature, glutamine is also essentially supplemented in culture medium to maintain cell survival and proliferation in experimental settings [29,30]. With the increasing focus on glutamine consumption in immune cells and its involvement in regulating immune functions [23], a specific glutamine-starved microenvironment has recently been linked with disrupted M2 polarization or increased proinflammatory cytokines (IL-6, IL-8, etc.) production in macrophages [18,31]. We also confirmed in this study that IL-4 treatment is rather weak in provoking an M2 phenotype switch in glutamine-deprived murine macrophages, although glutamine itself cannot induce M2 polarization independently. These results thus suggest an indispensable role of glutamine supply in the metabolic regulatory circuit rather than as the trigger of macrophage M2 polarization. Importantly, glutamine did not affect M1 polarization or promote proliferation of naïve macrophages, suggesting that glutamine may selectively promote the M2 polarization of macrophages. Two recent studies reported increased transport of extracellular glutamine [15] or accelerated intracellular glutamine catabolism [11] in macrophages upon IL-4 stimulation. Our current study also demonstrated that both extracellular glutamine deprivation and intracellular inhibition of glutamine synthesis could inhibit M2 polarization in IL-4 treated macrophages. These findings together indicate that IL-4 may not only upregulate the canonical transcriptional events that drive the expression of M2 genes but may also link glutamine uptake with metabolic adaptation that supports macrophage M2 activation.

Given that glutamine is extremely important in supporting M2 polarization in a direct metabolic manner, we next addressed whether glutamine exerts regulatory activities by affecting key modulators in the metabolic circuit of macrophages. PDH is a gatekeeper metabolic hub in the TCA cycle that irreversibly catalyses the conversion of pyruvate into acetyl-CoA, thereby driving the metabolic flux into OXPHOS [20,23]. Notably, the activity of PDH is impaired in M1 macrophages but maintained or even increased in M2 macrophages, suggesting that PDH may serve as a critical target for the metabolic regulation of phenotypic switching in macrophages [12,21]. In this study, we demonstrated that glutamine enhances the activity of PDH. Moreover, the ability of glutamine to augment macrophage M2 polarization is remarkably suppressed by siRNA knockdown or pharmacological inhibition of PDH. Several recent studies have revealed that glutamine and its metabolites may play moonlighting regulatory roles other than directly providing intermediate substrates that replenish the TCA cycle. For example, glutamine facilitates the demethylation of histone H3K27 to upregulate M2 gene expression [10] or activates the uridine diphosphate *N*-acetylglucosamine pathway that mediates the glycosylation of M2 marker proteins [18]. Additionally, according to a labelling study, only one-third of all carbons in TCA cycle metabolites are derived from glutamine in M2 macrophages [18]. In this regard, it is reasonable that glutamine may also regulate metabolic checkpoints to maintain an intact OXPHOS process, in addition to its direct effect by providing αKG that fuels the TCA cycle [10]. Furthermore, our results may couple with previous findings to synergistically reveal a profound regulatory machinery consisting of metabolic, transcriptional and posttranslational mechanisms, which are employed by glutamine to orchestrate macrophage M2 polarization.

Multifaceted regulatory patterns, including transcriptional regulation and posttranslational modification, have been identified to control the enzyme activity of PDH [20,22]. Typically, fine-tuning regulation of PDH activity has been extensively demonstrated by phosphorylation and dephosphorylation of its key subunit PDHA1, which is controlled mainly by PDKs and PDH phosphatases, respectively [32]. Unexpectedly, we found that neither PDH expression nor a primary phosphorylation status (Ser293) on PDH is altered in IL-4-treated macrophages regardless of glutamine supplementation. These results strongly imply that glutamine promotes PDH activation with noncanonical protein modification mechanisms other than upregulating its expression or affecting its protein phosphorylation. According to a previous protein complex analysis, the lysine residues on PDH can also be modified by acetylation and succinylation, both of which affect its activity [20]. Although we did not observe a change in protein panacetylation upon glutamine supplementation (Figure S4), we found that glutamine sustains the succinylation of PDH by repressing the expression and desuccinylase activity of SIRT5, thereby uncovering a new role of glutamine in modulating protein desuccinylation by targeting a key lysine desuccinylase. SIRT5 is a mitochondrial resident sirtuin that catalyses the removal of negatively charged lysine acyl modifications, including succinylation [33]. Accumulated data have documented SIRT5 as a critical regulator of metabolic proteins involved in glycolysis, OXPHOS and fatty acid oxidation [33]. An earlier study even demonstrated that SIRT5 could desuccinylate PDH and repress its biochemical activity in mouse embryonic fibroblasts [22]. In support of this result, our study similarly indicates that glutamine represses SIRT5 expression and disrupts its desuccinylation activity, thereby sustaining PDH activity and controlling M2 polarization. Moreover, our results are similar to those in a recent study focusing on SIRT5 and the immune microenvironment in hepatocarcinogenesis [34]. In this study, the authors also described that downregulation of SIRT5 leads to abnormal bile acid metabolism and favours M2-like tumour-associated macrophages. Notably, SIRT5 has no detectable or rather weak deacetylase activity [35], which is consistent with our findings in this study that protein acetylation is unaffected by glutamine. However, SIRT5 may catalyze the removal of mutiple acyl modifications, such as malonylation, succinylation or glutarylation, on the lysine residues of proteins [35,36]. In this regard, future work is warranted to depict a more precise picture of protein modification profiles driven by SIRT5 under the regulation of glutamine.

Glutamine is catabolized into glutamate and then into αKG and succinyl CoA to fuel the TCA cycle [10]. By using a chemical inhibitor that interferes with different stages of glutamine metabolism, we found that glutamine is most likely to take effect via its catabolism into αKG but not via further metabolites of succinyl-CoA. It has been considered that αKG displays an anti-inflammatory nature. In addition, a recent study by Liu *et al*. confirmed that αKG enhances M2 polarization of murine macrophages [10]. Similarly, our subsequent results also demonstrated that direct supplementation with a membrane-permeable analogue of αKG could replace glutamine to promote M2 polarization. However, Liu *et al*. showed that a high αKG/succinate ratio favours jumonji domain-containing 3-dependent histone demethylation, thereby directly upregulating the transcription of M2 genes. Our study may reveal another metabolic regulatory effect of αKG that interferes with the desuccinylation of PDH, which indirectly supports M2 polarization with energy demand relying on sustained OXPHOS. Notably, our results do not support the requirement for conversion from αKG to succinyl-CoA, which represses SIRT5 and sustains PDH activity. This result seems contradictory to our main finding that PDH succinylation maintains its ability to drive M2 polarization. However, previous findings have demonstrated that propionyl CoA produced by fatty acid β-oxidation can replenish the TCA cycle by transforming into succinyl CoA, suggesting an alternative route of succinyl CoA anaplerosis rather than αKG conversion [37–39]. Additionally, αKG can couple with the αKG dehydrogenase complex to drive enzyme-dependent succinylation of PDHA1 in the absence of succinyl-CoA, as shown by a previous study performed in neurons [40]. These results thus support our findings that αKG is essential for maintaining succinylation of PDH even when the routine circuits for succinyl-CoA generation are interrupted.

Glutamine has been traditionally used in treating burns and postburn syndromes, with underlying mechanisms involving alleviating hypermetabolism, promoting energy synthesis and restoring intestinal mucus barrier function, as demonstrated by our previous work [41–44] and that of others [25,29,45,46]. However, it is not known whether glutamine is beneficial for burn sepsis due to its ability to augment macrophage M2 polarization. By using a murine burn sepsis model, we confirmed that glutamine alleviates burn sepsis injury by promoting M2 polarization. Although we only observed a tendency of survival improvement, due to the limited number of animals and the complexity of sepsis treatment which cannot be achieved by the independent use of therapeutic agents, such as glutamine, our study demonstrated a well-defined liver-protective effect of glutamine. Importantly, these effects were all repressed when macrophage M2 polarization was disrupted, suggesting that glutamine alleviates burn sepsis injury via promoting M2 polarization in macrophages. In earlier studies, glutamine-mediated M2 polarization was shown to play multifaceted roles, such as preventing obesity/type 2 diabetes, delaying the process of atherosclerotic cardiovascular diseases or otherwise promoting tumour growth [14,16,47,48]. The present study not only confirms a fundamental effect of glutamine in supporting M2 polarization but may also offer new insight into its pharmacological efficacy in treating burns and postburn complications.

## Conclusions

In summary, we reveal a new role of glutamine in augmenting M2 polarization in IL-4-treated murine macrophages by modulating protein modification of the metabolic checkpoint enzyme PDH. Specifically, this performance is mediated by the glutamine-derived metabolite αKG, which suppresses SIRT5 expression and its desuccinylation ability on PDHA1, thereby sustaining PDH activation to allow an intact OXPHOS process required for M2 polarization. Furthermore, our study may strengthen the pharmacological efficacy of glutamine in treating burns by showing its ability to prevent excessive inflammation and reduce organ damage by promoting macrophage M2 polarization in a murine burn sepsis model.

## Abbreviations

ALT, Alanine transaminase; Arg1, Arginase-1; AST, Aspartate aminotransferase; BIFC, Bimolecular fluorescence complementation; BMDMs, Bone marrow derived macrophages; CO-IP, Co-immunoprecipitation; DM-αKG, Dimethyl α-ketoglutarate; DMEM: Dulbecco’s modified eagle’s medium; ELISA, Enzyme-linked immunosorbent assay; FBS, Fetal bovine serum; Fizz1, Found in inflammatory zone 1; Gln, Glutamine; IL-4, Interleukin 4; L-α-A, L-α-Aminoadipic acid; Jmjd3, Jumonji domain-containing 3; αKG, α-Ketoglutarate; LPS, Lipopolysaccharide; M-CSF, Macrophage colony-stimulating factor; NS, Normal saline; OE, Overexpression; OXPHOS, Oxidative phosphorylation; Pan-succK, pan-succinyllysine; Pan-acetK, pan-acetyllysine; PCNA, Proliferating cell nuclear antigen; PDH, Pyruvate dehydrogenase; PDHA1, Pyruvate dehydrogenase E1 subunit alpha 1; p-PDHA1, Phosphorylated PDHA1; PDK: PDH kinases; PLF, Peritoneal lavage fluid; SIRT5, Sirtuin 5; SP, Succinyl phosphonate; TCA, Tricarboxylic acid; TNF-α, Tumor necrosis factor α.

## Funding

This research was funded by National Natural Science Foundation of China (82 172 202 and 81 902 015). Innovative Leading Talents Project of Chongqing (NO. CQYC20210303286).

## Authors’ contributions

XP and XL conceived and designed the study. YZ, XC, YL and LX executed experiments and collected data. SF and QH analysed the data; YZ, XC and LX drafted and formatted the manuscript. XP corrected the manuscript. All authors have read and agreed to the published version of the manuscript.

## Ethics approval and consent to participate

All mice were housed under standard pathogen-free conditions with free access to water and food. Animal experiments were approved by the Institutional Animal Ethics Committee of Third Military Medical University and performed in accordance with the national guidelines for animal welfare (No. AMUWEC20201429).

## Conflicts of interests

None declared.

## Supplementary Material

Supplementary20220718_tkac041
