# Supplementary material for: Glutamine mitigates murine burn sepsis by supporting macrophage M2 polarization through repressing the SIRT5-mediated desuccinylation of pyruvate dehydrogenase
Source: Burns Trauma. 2022 Dec 30;10:tkac041. doi: 10.1093/burnst/tkac041 (PMC9801296; doi:10.1093/burnst/tkac041)
Supplement: Supplementary20220718_tkac041 [file supplementary20220718_tkac041.docx]

**Supplementary Materials**

**Table S1** **Sequence of PCR primers**

| **Name** | **Primer Sequence** |
| --- | --- |
| Arg1 | F: 5’-CTCCAAGCCAAAGTCCTTAGAG-3’  R: 5’-GGAGCTGTCATTAGGGACATCA-3’ |
| Fizz1 | F: 5’-CCAATCCAGCTAACTATCCCTCC-3’  R: 5’-ACCCAGTAGCAGTCATCCCA-3’ |
| Ym1 | F: 5’-CAGGTCTGGCAATTCTTCTGAA-3’  R: 5’-GTCTTGCTCATGTGTGTAAGTGA-3’ |
| SIRT5 | F: 5’-CCAGTTGTGTTGTAGACGAAAGC-3’  R: 5’-TTCCGAAAGTCTGCCATATTTGA-3’ |
| PDHA1 | F: 5’-TGTGACCTTCATCGGCTAGAA-3’  R: 5’-TGATCCGCCTTTAGCTCCATC-3’ |
| β-actin | F: 5’-GGCTGTATTCCCCTCCATCG-3’  R:5’-CCAGTTGGTAACAATGCCATGT-3’ |

**
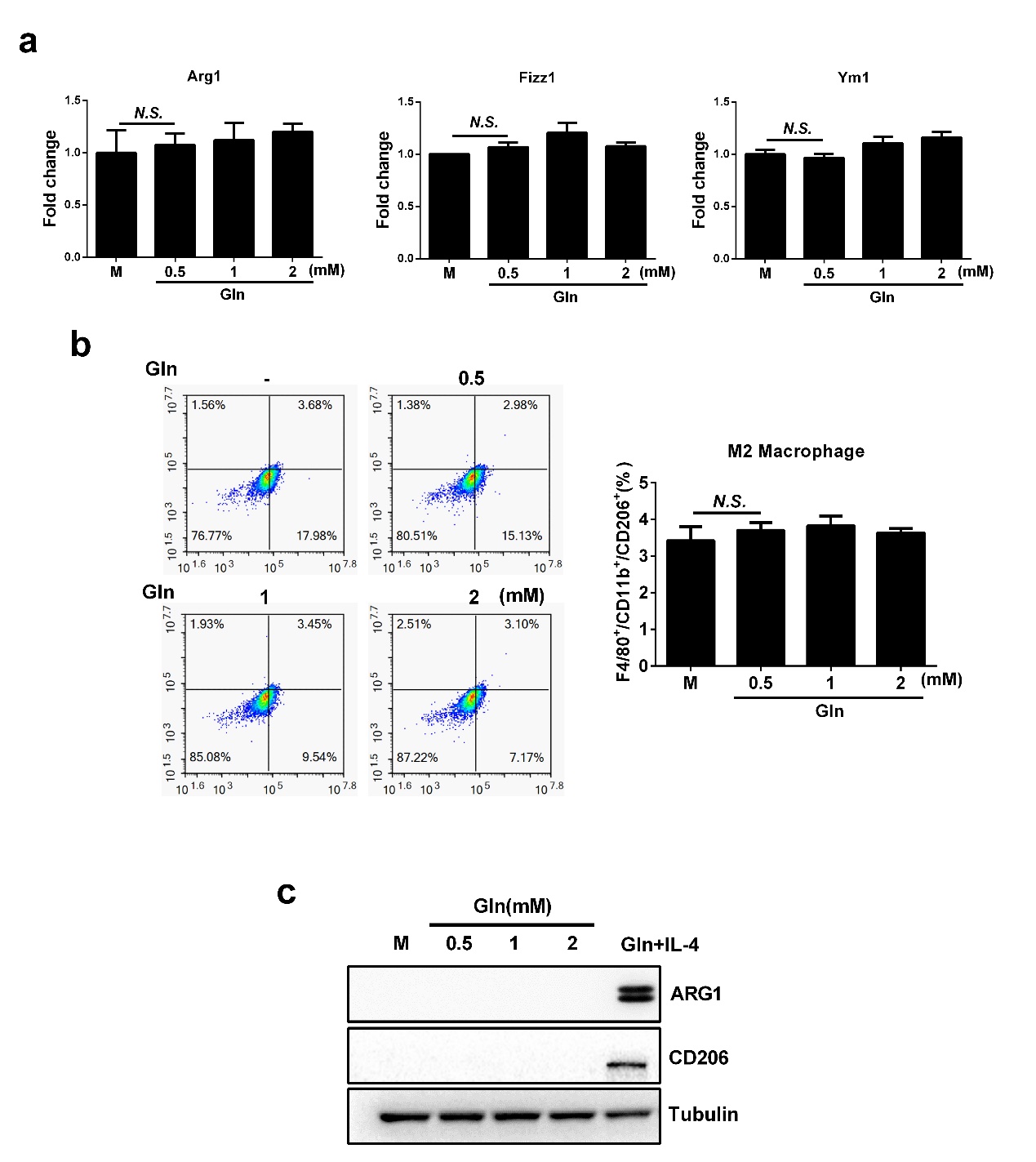
**

**Fig. S1** **Glutamine alone does not induce M2 polarization of macrophages.** (a) Murine BMDMs were incubated in DMEM with gradient concentration of glutamine (Gln, 0.5-2 mM) for 24 h. The mRNA expression of Arg1, Fizz1, Ym1 was detected by RT-PCR (a). F4/80^+^/CD11b^+^/CD206^+^ cells were detected by flow cytometry (b). (c) BMDMs were treated with glutamine (0.5-2 mM) or glutamine with 50 ng/ml IL-4 for 24 h. The protein expression of Arg1 and CD206 was detected by western blot (c). *N.S.*: no significance. n = 3. Glutamine abbreviated as Gln.


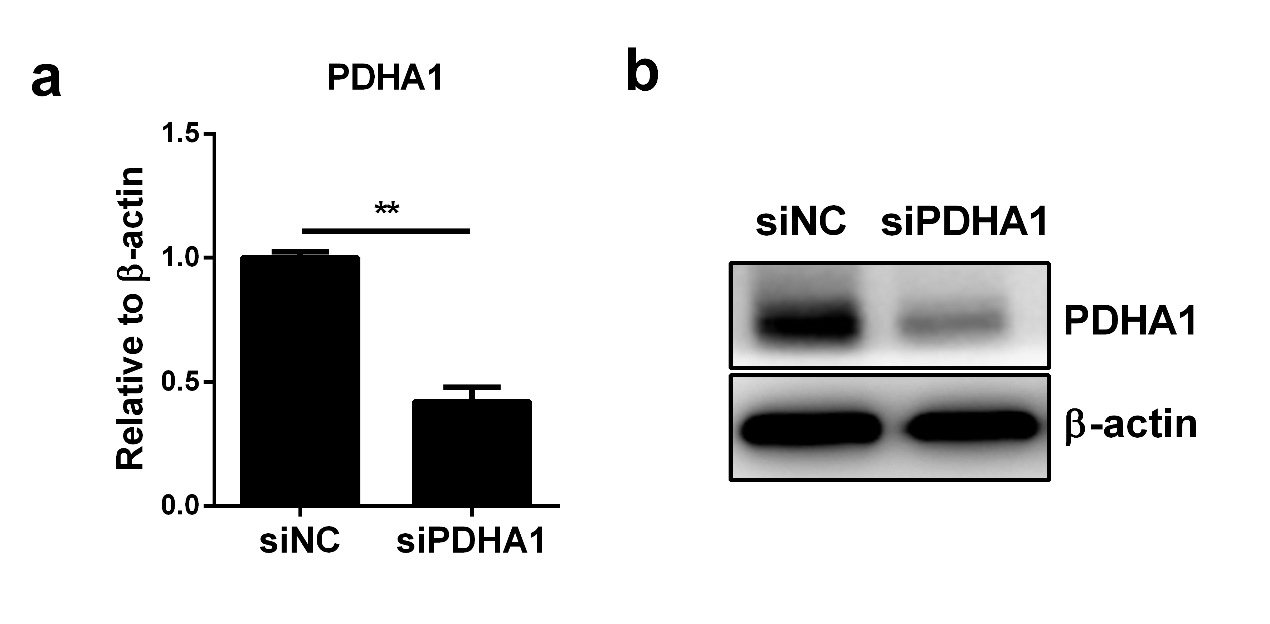


**Fig. S2 The mRNA and protein expression of PDHA1 is downregulated by siRNA interference.** J774A.1 cells were transfected with control siRNA (siNC) or PDHA1 siRNA (siPDHA1) for 48 h and the mRNA and protein expression of PDHA1 was detected by RT-PCR and western blot, respectively.


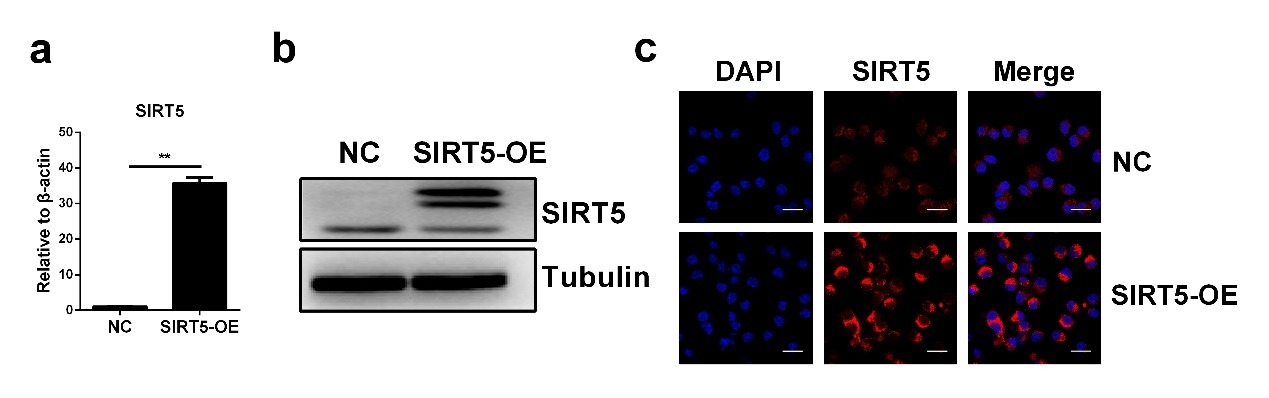


**Fig. S3 Overexpression of SIRT5 by lentivirus transfection**. (a-c) J774A.1 cells were infected with vehicle (NC) or SIRT5 overexpresses lentivirus (SIRT5-OE) for 72 h, and then the cells were screened by 5 µg/ml puromycin for 24 h. The mRNA expression of SIRT5 was detected by RT-PCR (a). The protein expression of SIRT5 was detected by western blot (b) and immunofluorescence (c). **: P<0.01. Bar: 20 µm. n = 3.


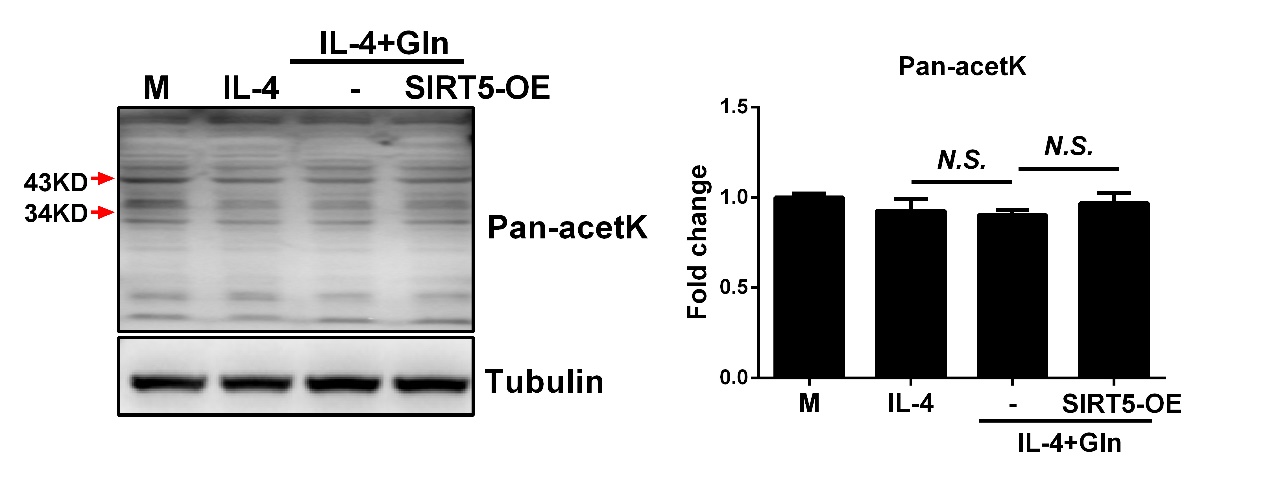


**Fig. S4 Effect of Glutamine on protein pan-acetylation modification.** J774A.1 cells were infected with vehicle or SIRT5 overexpresses lentivirus (SIRT5-OE), and then further treated with 50 ng/ml IL-4, or IL-4 with 2 mM glutamine. The pan-acetyllysine (Pan-acetK) was detected by western blot. *N.S.*: no significance. n = 3. Glutamine abbreviated as Gln.
